# Immunoregulatory functions and the therapeutic implications of GARP-TGF-β in inflammation and cancer

**DOI:** 10.1186/s13045-018-0570-z

**Published:** 2018-02-20

**Authors:** Alessandra Metelli, Mohammad Salem, Caroline H. Wallace, Bill X. Wu, Anqi Li, Xue Li, Zihai Li

**Affiliations:** 1 Department of Microbiology and Immunology, Hollings Cancer Center, Medical University of South Carolina, Charleston, SC 29425 USA; 2 Children’s Hospital Boston, Harvard Medical School, Boston, MA 02115 USA; 3 The First Affiliated Hospital, Zhengzhou University School of Medicine, Zhengzhou, 450052 China

**Keywords:** TGF-β, GARP, Treg, Immune tolerance, Cancer, Platelets

## Abstract

GARP (glycoprotein-A repetitions predominant) is a type I transmembrane cell surface docking receptor for latent transforming growth factor-β (TGF-β) that is abundantly expressed on regulatory T lymphocytes and platelets. GARP regulates the availability of membrane-bound latent TGF-β and modulates its activation. For this reason, GARP expression on immune and non-immune cells is involved in maintaining peripheral tolerance. It plays an important role in preventing inflammatory diseases such as allergy and graft versus host disease (GvHD). GARP is also frequently hijacked by cancer cells to promote oncogenesis. This review summarizes the most important features of GARP biology described to date including gene regulation, protein expression and mechanism in activating latent TGF-β, and the function of GARP in regulatory T cell biology and peripheral tolerance, as well as GARP’s increasingly recognized roles in platelet-mediated cancer immune evasion. The promise for GARP-targeted strategy as a novel immunotherapy of cancer is also highlighted.

## Background

Transforming growth factor-β (TGF-β) is a pleiotropic cytokine expressed by the majority of cells and found in all tissues [1]. It plays important roles in numerous aspects of biological processes such as cell proliferation, development, apoptosis, fibrosis, angiogenesis, wound healing, cancer, and much more [2–4]. TGF-β’s production and secretion consist of a series of tightly regulated steps, as any dysregulation can lead to disease [5, 6]. Biochemically, TGF-β exists in at least four different forms: (1) freely soluble TGF-β; (2) soluble TGF-β associated with latency-associated peptide (LAP), known as latent TGF-β (LTGF-β); (3) TGF-β-LAP-LTBP, latent TGF-β associated with latent TGF-β-binding protein (LTBP); and (4) membrane-associated latent form of TGF-β [7, 8]. Three isoforms of TGF-β exist, TGF-β1, TGF-β2, and TGF-β3, encoded by three different genes; yet, TGF-β1 is the most studied among the three [9, 10].

Glycoprotein-A repetitions predominant protein (GARP) has emerged as a critical regulator of latent TGF-β activation [11–14]. By binding to LTGF-β, GARP acts as a docking receptor that concentrates LTGF-β on the cell surface and enhances its final activation [15]. The function of GARP has been extensively studied on regulatory T lymphocytes (Tregs), where it complexes with αVβ8 integrins to release active TGF-β from the surface of the cells [16, 17]. Via this function, GARP was shown to be involved in enhancing the suppressive phenotype of Tregs and in maintaining Treg-mediated peripheral tolerance [12, 18, 19].

**Fig. 1:**
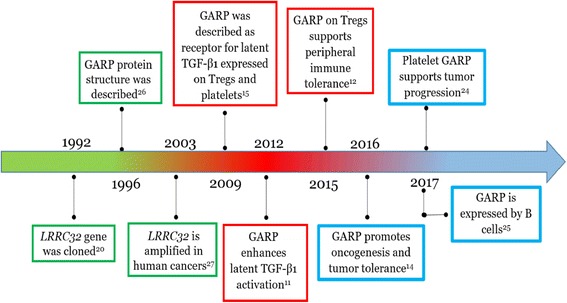
Timeline of literature on the study of GARP. GARP literature can virtually be divided into three time windows; each emphasizes interests of the field in a specific aspect of GARP function: the first round (green) of research focused on the characterization of the gene and the protein structure; the second round (red) was dedicated to studying the GARP function on Treg cells and tolerance; research during the latest period (light blue) analyzes GARP expression and function on platelets and cancer. The most pioneristic articles are indicated

Since the discovery of GARP in 1992 [20], the scientific literature regarding GARP can be divided into three consecutive time periods; each of them emphasizes research on a specific aspect of the protein (Fig. 1). Initially, GARP gained attention between 1992 and 2006 because of its gene amplification in aggressive forms of human metastatic carcinomas [21–23]. Subsequently, GARP was identified as a latent TGF-β1 receptor expressed on immune cells, specifically on Tregs and megakaryocytes/platelets [15]. At this time, GARP was identified as a Treg activation marker and for its ability to regulate the bioavailability of TGF-β [11]. During the last 3 years, several new aspects of GARP have been discovered. For example, our recent findings established a strong connection between GARP and cancer by describing the pro-tumorigenic function of this protein in several human malignancies [14] and the unexpected role of platelet GARP in immune evasion and the cancer progression [24]. Moreover, GARP expression has been recently described on human B cells in response to B cell receptor activation and Toll-like receptor (TLR) 9 ligation [25] (Caroline Wallace and Zihai Li, unpublished).

## GARP: gene and protein structure

### *Lrrc32* gene

In their studies on gene amplification, Ollendorff and colleagues identified a new independent unit designated D11S833E in the telomeric region at 11q13-q14, which they named GARP [20, 26]. In situ hybridization studies revealed that the murine *Lrrc32* gene is localized on chromosome 7 in a region that is conserved between human and murine genome [20]. Interestingly, *Lrrc32* gene locus is part of a chromosomic region frequently altered in human cancers [27]. Indeed, *Lrrc32* specific gene amplification was observed in human breast cancer [28] and primary and metastatic neck lymph nodes in oral squamous cell carcinoma [22]; moreover, in prostate cancer, *Lrrc32* amplification rate increases with the decrease of hormone sensitivity [23]. Conversely, deletion and rearrangement of *Lrrc32* locus were described in two cases of hibernoma, thus unveiling the ambiguous behavior of *Lrrc32* gene product in human malignancies [29]. Nucleotide blasting analysis showed that human and murine *Lrrc32* gene have a similar structure, i.e., they share 81% of homology and both comprise of two coding exons; in particular, the first exon encodes for the signal peptide and nine amino acids, and the second exon encodes for the majority of the coding region [30].

### GARP gene regulation

Upon T cell receptor (TCR) engagement, GARP expression is induced in Tregs; no significant surface expression of GARP has been described in human or mouse conventional T helper (Th) cells [31]. Haupt and colleagues discovered that cell and context-specific expression of the GARP gene is the result of the interplay of two alternative promoters: upstream promoter 1 (P1) and downstream promoter 2 (P2). Both promoters drive GARP gene transcription; however, the variance in their methylation status in different cell populations dictates where, and under what conditions, GARP will be expressed. P2 is almost completely demethylated in both Tregs and Th cells, yet only in Th cells is the transcription initiation from P2 blocked by several methylated CpG islands present in the downstream P1. Also, by inhibiting binding with transcription factors, the methylated CpGs maintain P1 in a closed chromatin configuration. In contrast, the less pronounced methylation status of P1 in Tregs allowed the binding of its nuclear master transcription factor forkhead box P3 (FoxP3) that remodels the promoter region toward an open configuration status. This allows the subsequent binding of nuclear factor of activated T cells (NFAT) and nuclear factor-κB (NF-κB) to drive the transcription of the GARP gene [32]. A clear example of this FoxP3-mediated GARP expression is the conversion of tumor-specific Th17 cells to ex-Th17 FoxP3^+^ cells that show upregulation of surface GARP as a transdifferentiation-associated marker [33]. Accordingly, knocking-down of FoxP3 with RNA interference in Tregs reduced surface GARP, yet GARP silencing did not affect FoxP3 expression [34]. Tregs are not the only cell population that experiences GARP-FoxP3 co-regulation; human and murine megakaryocytes and platelets constitutively express both FoxP3 and the surface GARP/LAP complex. Interestingly, upon activation, platelets upregulate both GARP and FoxP3: protease-activated receptor 4 activating peptide (PAR4-AP) increases surface GARP, while phorbol ester myristate acetate upregulates FoxP3 expression [15, 35, 36]. Although the simultaneous upregulation of GARP and FoxP3 needs to be demonstrated, these findings suggest that platelets are another subset of cellular entities where GARP and FoxP3 interdependence might occur. In addition to lymphocytes and platelets, human melanocytes simultaneously express FoxP3 and GARP [37], further demonstrating this association.

Even though many reports are in favor of FoxP3 and GARP co-expression, other reports suggest that expression of FoxP3 and GARP is independent of each other. For example, while FoxP3 shRNA affects GARP expression, GARP shRNA does not change FoxP3 expression in expanded Tregs [34]. Furthermore, Helios, but not FoxP3, has been described as the marker of activated Tregs expressing GARP/LAP [38]. Additionally, GARP is inducible on activated human CD19^+^CD20^+^ B cells through B cell receptor (BCR) and TLR 9 engagement by anti-immunoglobulin (Ig) M antibodies and unmethylated bacterial DNA (CpG), respectively, where it enhances class switching recombination and production of IgA [25] (Caroline Wallace and Zihai Li, unpublished). Based on this last observation, it is reasonable to hypothesize that TLR signaling induces GARP expression via NF-κB, since GARP promoter has a putative NF-κB binding region as previously mentioned. All these reports indicate that the interdependence of GARP and FoxP3 expression remains an intriguing area that is far from being completely understood [38].

In addition to FoxP3, signal transducer and activator of transcription 3 (STAT3) is another transcription factor that was found recently to regulate GARP gene expression; Interleukin (IL)-6 administration to CD4^+^ naïve T cells is sufficient to restrain GARP transcription and expression via the STAT3 signaling pathway [39]. As will be discussed later, GARP is a latent TGF-β receptor that enhances furin-mediated pro-TGF-β cleavage, yet the expression of GARP per se is independent of both TGF-β and Furin [40].

Post-transcriptional regulation is another important checkpoint in GARP expression. The distal part of the 3′ untranslated region (UTR) of GARP transcript is targeted by six microRNAs (miRNAs) which decrease GARP protein expression. Among these six microRNAs, miR-142-3p is expressed 2.5 times more in Th cells than in Tregs, and upon TCR stimulation, miR-142-3p expression decreases in both T cell populations [41]. MiR-142-3p facilitates the formation of a complex that together with argonaute 2, and GARP-mRNA controls GARP expression via post-transcriptional regulation [42].

### GARP protein

**Fig. 2:**
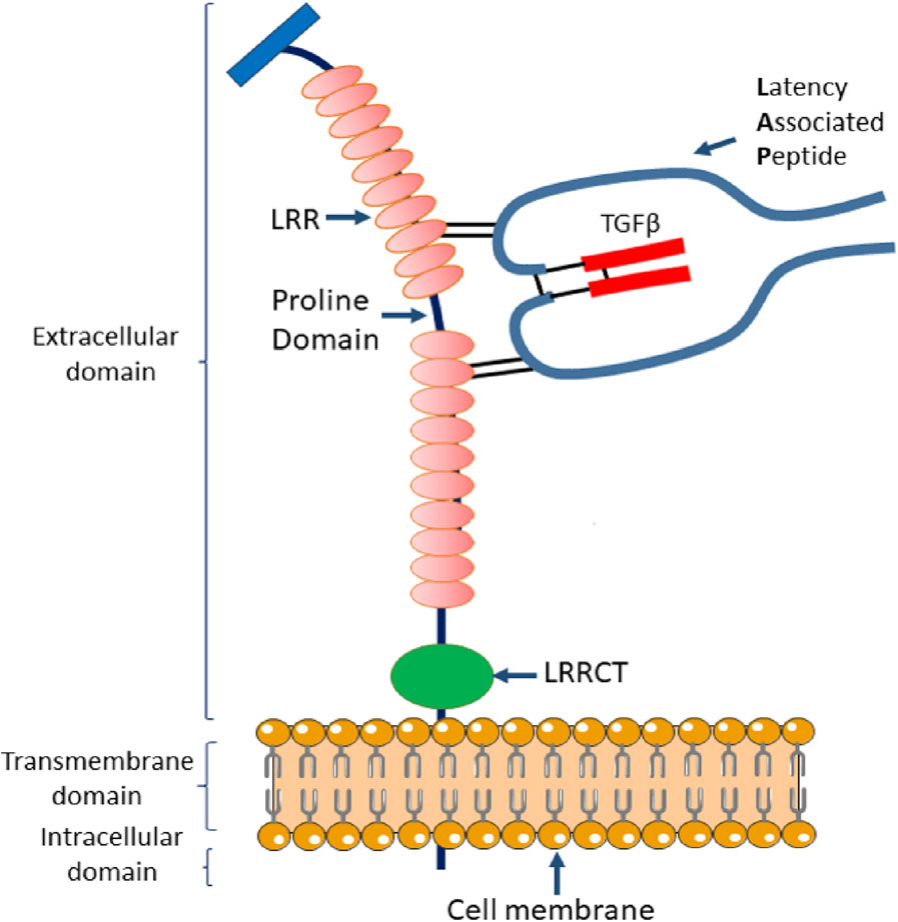
Structure of the membrane-bound GARP-latent TGF-β1 complex. GARP protein is structurally divided into three domains based on its primary sequence: the extracellular domain, the transmembrane domain, and the intracellular domain. The extracellular domain contains two sets of ten LRRs divided by a proline-rich domain and one C-terminal LRR (LRRCT). Two conserved Cys residues (Cys-192 and Cys-331) are located on the 7th and 12th LRR, respectively, and are responsible for two disulfide bond formation between GARP and Cys-4 of LAP of latent TGF-β

After gene isolation, human and mouse GARP protein putative sequences were deciphered. GARP protein is a type I transmembrane protein and can be divided into three domains: the extracellular domain, which constitutes approximately 70% of the protein; the hydrophobic transmembrane domain; and the 15 amino acid residue cytoplasmic tail (Fig. 2). As part of the leucine-rich repeats (LRR)-containing proteins family, the extracellular domain of GARP contains 20 LRR motifs, divided into two groups by a proline-rich region, and a C-terminal LRR (LRRCT) [26, 30]. Among the extracellular LRR proteins, GARP, together with TLRs, glycoprotein 1bα and glycoprotein 1bβ, belongs to the LRR Tollkin subfamily, a group of proteins involved in inflammation [43]. Like TLRs, Zhang et al. showed that GARP requires the master chaperone gp96 (GRP94) in the endoplasmic reticulum for its folding and surface expression [44, 45]. The proline-rich region located between the LRR of GARP resembles the hinge domain of the LTBP. This domain confers flexibility to the protein and suggests that GARP might be involved in protein-protein interaction [46, 47]. Additionally, like LTBP, GARP covalently disulfide links with LAP; site-specific mutagenesis from Cys to Ala demonstrated that Cys-192 and Cys-331, located on the 7th and 12th LRR respectively, are responsible for the disulfide linkage between GARP and Cys-4 of LAP [11]. Despite the high homology in the extracellular domain, murine and human cytoplasmic tails show a 33% difference in the amino acid sequence, yet they both have a conserved tyrosine residue. Of interest, other members of LRR_Tollkin family, like TLRs, have a cytoplasmic phosphorylated tyrosine involved in signal transduction, suggesting a possible tyrosine phosphorylation-dependent function for GARP [48].

## Tissue distribution and cell type-specific expression

In human tissues, GARP is expressed in the peripheral blood, placenta [49], and pancreas [50]. In accordance with the mRNA expression data, GARP protein is expressed by human breast cancer, lung cancer, and colon cancer cells, where higher GARP expression correlates with worse clinical outcome [14]. GARP expression has been reported in multiple human and mouse cells—specifically on human activated B cells [25] (Caroline Wallace and Zihai Li, unpublished), human and mouse mesenchymal stromal cells [51], Tregs [15], megakaryocytes/platelets [52], hepatic stellate cells [53], and mouse LAP^+^ γδ T cells [54]. GARP is widely expressed in the mouse lymphoid organs, including the spleen, the mesenteric and peripheral lymph nodes, and the thymus as well as the Peyer’s patches [40]. This is not surprising because GARP-expressing Tregs are abundantly present in these sites. We recently demonstrated that GARP is strategically expressed on the medial edge epithelial cells of the palate shelf during embryogenesis, where it is critical for TGF-β3 activation and signaling, and is thus indispensable for normal palatogenesis [55]. We showed that whole-body *Lrrc32-*null mice do not survive 24 h after birth as a result of the defect in the fusion of the palatal shelves, a phenotype indistinguishable from the *Tgfb3*-null mice [56].

The presence of soluble GARP as a result of shedding from T cell membrane has also been reported [41]. The possibility of a shedding process was first discussed by Roubin and colleagues in 1996 when, describing a GARP deduced amino acid sequence, they observed the presence of a hydrophobic leader sequence. They hypothesized that this domain might be the signal peptide for targeting the protein to the secretory pathway [30]. Soluble GARP indeed is present in human plasma [57], yet the mechanism and significance of the protein’s shedding or secretion is not clear.

## GARP function in TGF-β maturation and activation

GARP is expressed on the cell surface where it was thought initially to be the docking receptor only for latent TGF-β1 [58]. Of interest, the association with GARP is not uniquely limited to latent TGF-β1; latent TGF-β2 binds to GARP with a much lower binding affinity [15]. Importantly, the genetic and biochemical evidence demonstrated that GARP is absolutely required for the association to and activation of latent TGF-β3 [55]. Thus, GARP can bind to all three TGF-β isoforms.

**Fig. 3:**
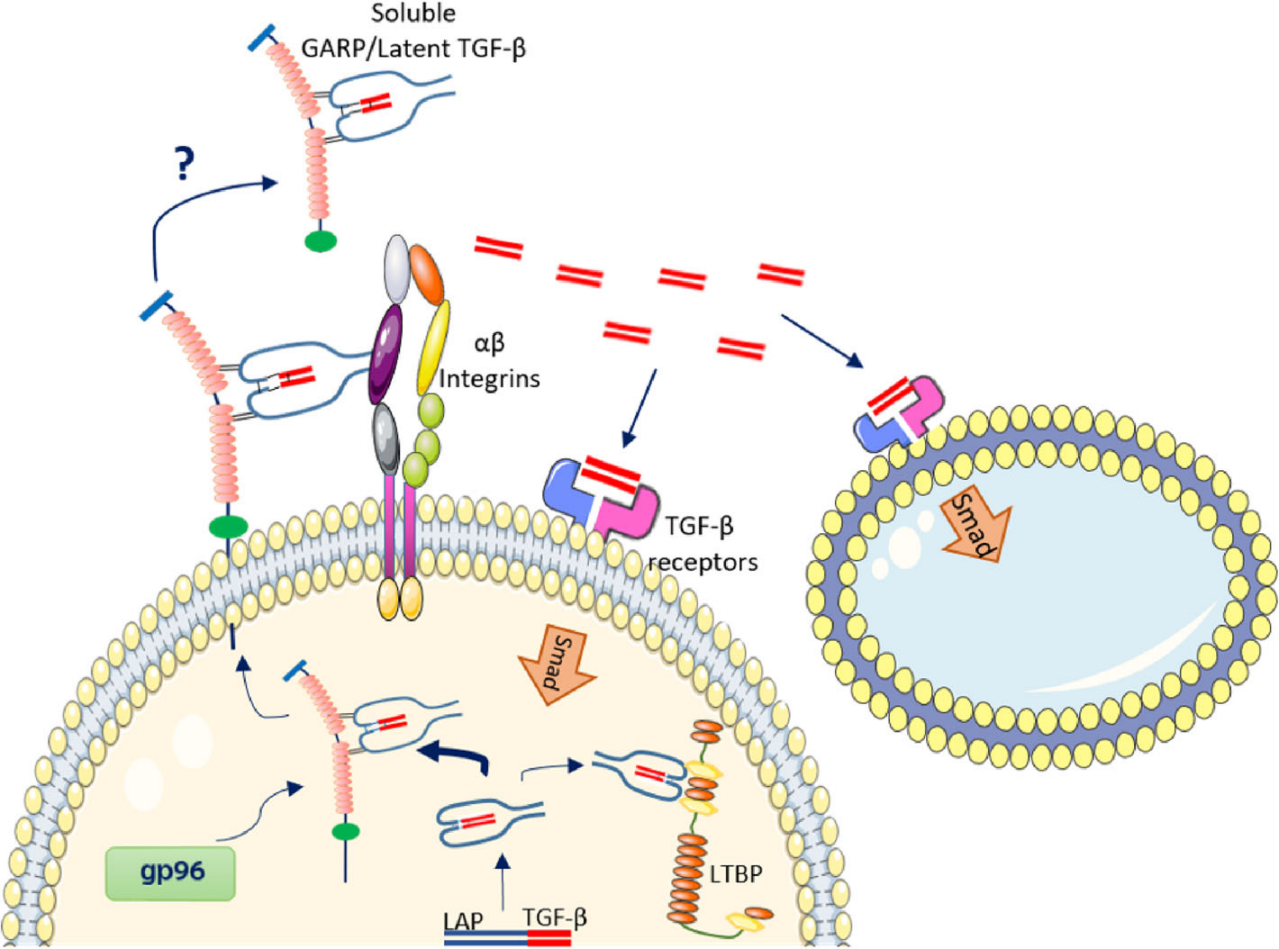
GARP functions in TGF-β maturation and activation. TGF-β is synthetized as an inactive homodimeric pro-protein that is cleaved by a furin-like protease to yield the formation of latent TGF-β. GARP enhances furin-dependent cleavage and associates with latent TGF-β. The master chaperone gp96 in the lumen of the endoplasmic reticulum (ER, not depicted) ensures the proper folding of GARP and its surface expression. On the cell surface, GARP/latent TGF-β complex associates with alpha-beta integrins (αVβ6 and αVβ8) to release the mature TGF-β peptide. Mature TGF-β interacts with TGF-β receptors on the cell surface in both an autocrine and paracrine fashion. In some cases, GARP/latent TGF-β complex can also be released from the cell surface, but how TGF-β is activated from the soluble complex is not clear

As a very powerful cytokine, sometimes referred to as a “beast” [59], TGF-β production and secretion consist of multiple tightly regulated steps; interestingly, GARP plays a role in each of them (Fig. 3). First, TGF-β is synthesized and secreted through the secretory pathway as inactive homodimeric pro-proteins that are cleaved by furin-type proteases to generate a mature TGF-β. At this stage, the newly synthetized molecule is both covalently (through disulfide bonds) and non-covalently associated with LAP, referred to as latent TGF-β [10]. A study from Sophie Lucas’ laboratory demonstrated that GARP increases the rate of pro-TGF-β cleavage in a furin-independent manner [41].

Subsequently, latent TGF-β associates with the LTBP, creating the large latent complex (LLC) [60]. GARP can interfere with this association due to its higher binding affinity to latent TGF-β; when both GARP and LTBP are co-expressed in 293 T cells, GARP outcompetes LTBP for latent TGF-β binding. Interestingly, electron microscopy analysis showed that GARP and latent TGF-β association also can be mediated by non-covalent binding [11]. The nature and function of this weakly associated complex might mediate TGF-β activation upon surface shedding [61], as discussed below.

Finally, the release of the biologically active mature TGF-β requires the separation and release of the mature form of TGF-β from the LAP. Multiple mechanisms have been evoked to describe this critical step, where cell surface integrins are the main orchestrators. αVβ6 and αVβ8 can activate latent TGF-β through proteases-dependent and protease-independent mechanisms. In the protease-independent manner, αVβ6 and αVβ8 integrins bind to the latent TGF-β and, after deforming the surface LAP, they mediate the release of the mature form of TGF-β. In the protease-dependent mechanisms, integrins recruit metalloproteinases or serine proteases that cleave LAP and, subsequently, liberate TGF-β [62, 63]. For example, thrombin mediates the activation of latent TGF-β bound to αVβ6 in a murine pulmonary injury model [64]. Recent findings demonstrated that membrane-bound GARP facilitates the protease-independent TGF-β activation via the formation of a complex together with αVβ6 integrins and latent TGF-β. Intriguingly, the association of the GARP/latent TGF-β complex with integrins does not disrupt the ring-like structure of the pro-TGF-β, suggesting that the integrin interaction is essential, yet it is not sufficient for secretion of mature TGF-β [11]. This may indicate that similarly to latent TGF-β alone, integrin binding to LAP predisposes the complex for the release of the active peptide; however, extra tensile force is required for the removal of the “straightjacket” elements of LAP [65]. This might explain why active TGF-β released from GARP/latent TGF-β complex is not always detectable, yet it is still able to activate TGF-β signal transduction, as shown in Tregs [12], B cells [25], and some TGF-β reporter cell lines [11].

Integrin contribution has also been described for activated Tregs; αVβ8 integrins are responsible for the release of latent TGF-β from the cell surface and for the formation of biologically active TGF-β as indirectly measured by Th17 induction [16]. Recent studies indeed demonstrated that on the Treg surface, GARP relies on the interaction with αVβ8 integrins to release active TGF-β [17]. Intriguingly, integrins and membrane tensile forces do not explain the release of mature TGF-β from soluble GARP (sGARP). This conundrum was partially unveiled by Fridrich and colleagues when they observed that mature TGF-β can be released from sGARP only when GARP and latent TGF-β are non-covalently associated [61]. However, the underlying mechanism is still obscure.

## GARP and peripheral immune tolerance

As discussed above, the GARP promoter has a binding site for FoxP3, indicating that Treg-specific transcription factor is required for GARP expression. Accordingly, silencing FoxP3 in human Tregs reduces surface GARP upon TCR stimulation [34]. On the other hand, enforced GARP expression in human Th cells endows cells with suppressive capability by upregulating several Treg and TGF-β signature genes including FoxP3, CD25, and CTLA4 [31, 66]. These findings suggest that the tolerogenic Treg phenotype might be reinforced by a positive feedback loop between GARP and FoxP3. Accordingly, in Treg cells that are differentiated in vitro, silencing of GARP partially impairs their suppressive ability [34]. In this regard, we demonstrated that mice lacking surface GARP on Tregs, due to a Treg-specific deletion of a molecular chaperone gp96, developed a fatal multi-organ inflammatory disease [44]. In these mice, indeed, GARP folding and surface expression were completely abrogated, thus preventing the formation of GARP/LTGF-β complex on activated Tregs and consequently the acquisition of the suppressive phenotype. Thus, gp96 deletion abolished the expression of cell surface GARP-LTGF-β as well as the mechanism of LTGF-β activation. Accordingly, this fatal phenotype can be partially rescued by exogenous active TGF-β administration [44]. It is noteworthy to mention that gp96 serves as a chaperone for numerous client proteins such as TLRs [67, 68] and multiple α and β integrin subunits [45, 69, 70], indicating that the inflammatory phenotype observed in Treg-specific gp96-deficient mice cannot be attributed to GARP deletion alone. Indeed, it has been recently shown that the final release of TGF-β from the GARP-LTGF-β complex requires the interaction of the complex with αVβ8 integrins, which are also chaperoned by gp96 [71].

The tolerogenic roles of GARP might give a mechanistic explanation for atopic dermatitis manifested by patients with gene mutations in the *Lrrc32* gene locus that prevents GARP surface expression [72]. Conversely, other reports indicate that FoxP3 is not required for GARP expression on Th cells upon TCR stimulation and that FoxP3^+^ Tregs maintain the same suppressive phenotype even in the absence of GARP [40].

The importance of GARP in peripheral tolerance is also indicated by the results of meta-analyses of genome-wide association studies which showed a strong correlation between *Lrrc32* gene locus expression and conditions like Crohn’s disease, ulcerate colitis [73], and allergic diseases [74]. In line with the findings of the genome-wide association studies, sGARP has been proven to be useful as an anti-inflammatory therapeutic agent by sustaining Treg immune-modulatory activity in a xenogeneic graft versus host disease (GvHD) model and in an allergen-specific gut inflammation system [19, 75]. In addition, allergic airway inflammation is mitigated by sGARP administration in a TGF-β-dependent manner [18]. On the other hand, Eschborn and colleagues showed that, while sGARP mitigates allergen-specific gut inflammation, injections of anti-GARP blocking antibody reduce the therapeutic effect of activated Tregs [75]. Additionally, blocking IL-6 signaling in the presence of TGF-β polarized Tregs to high GARP and LAP expression which are able to maintain oral tolerance in a delayed type hypersensitivity (DTH) model [39]. Furthermore, monoclonal GARP/latent TGF-β antibody blocks the autocrine production of active TGF-β in Tregs, restraining their immunosuppressive activity in a xenogeneic model of GvHD [12].

## GARP and cancer immune evasion

Although GARP offers an important protective role for the host in inflammation-driven pathological conditions, the tolerogenic FoxP3/GARP/TGF-β axis is a mediator of the immunosuppressive microenvironment that enhances tumor growth. For example, human ovarian cancer ascites are infiltrated with FoxP3^+^GARP^+^ Treg cells [33]. Higher frequency of GARP^+^FoxP3^+^ expression in Tregs positively correlates with an elevated immunosuppressive and more aggressive phenotype in advanced hepatocellular carcinoma [76].

As suggested by *Lrrc32* gene amplification, GARP protein is expressed on human cancer cells where it mediates the accumulation and subsequent activation of the circulating latent TGF-β [14]. GARP supports cancer cell growth and dissemination by providing an excellent reservoir of TGF-β that functions in the tumor microenvironment (TME) by regulating the innate and adaptive immune components and favoring tumor immune evasion. With regard to innate immunity, TGF-β inhibits natural killer (NK) cells cell and dendritic (DC) cells maturation [77, 78]. The role of TGF-β is also well studied in the non-resolving inflammation that facilitates cancer initiation [79, 80]. Tumor-derived TGF-β polarizes macrophages into tumor-associated macrophages (TAM) [81]. This cell population is a cancer therapeutic target because of its secretion of pro-inflammatory cytokines like IL-6, IL-23, and IL-17. Additionally, TGF-β derived from TAMs is one of the major drivers of the epithelial to mesenchymal transition [81–83]. Furthermore, tumor-derived TGF-β drives the formation of cancer-associated fibroblasts (CAFs), which in turn exert a strong pro-tumorigenic activity on epithelial cells by secreting their own TGF-β [84]. CAFs’ pro-tumorigenic properties have been reported for many malignancies such as prostate cancer [85], non-small cell lung carcinoma [86], and colorectal carcinoma [87]. Similarly, TGF-β impairs the adaptive anti-tumor immunity by directly inhibiting the clonal expansion and cytotoxicity of the CD8+ cytotoxic T cells (CTLs) [88, 89]. Finally, TGF-β indirectly attenuates CTLs by inducing the expression of Foxp3, which confers a regulatory and immune suppressive phenotype to CD4^+^ T cells [90].

Human malignant melanocytes express and secrete membrane-bound GARP and sGARP, respectively, that skew macrophages toward a polarized M2 phenotype and constrain the proliferation CTLs and their ability to produce cytokines [37]. In addition, GARP has been found to be highly expressed in human breast, colon, and lung cancers where GARP/TGF-β axis sustains primary tumor growth and distant metastasis. Intriguingly, a monoclonal antibody that blocks the binding between latent TGF-β and GARP was shown to be effective therapeutically in a murine syngeneic mammary carcinoma model [14]. Beyond blocking the GARP and latent TGF-β interaction on tumor cells, novel antibody-based strategies are emerging to block GARP/latent TGF-β complex on the surface of Tregs, thus preventing the secretion of active TGF-β. As reported above, a monoclonal human GARP antibody produced and tested in Sophie Lucas’ laboratory has been shown to inhibit the immunosuppressive activity of Tregs by blocking GARP in complex with latent TGF-β [12]. Although the efficacy of this antibody was tested on xenogeneic GvHD, the authors suggested that this antibody might be useful for cancer immunotherapy, a notion that remains to be tested. In line with this consideration, however, LAP blocking antibody has been proven to reduce tumor growth in animal models of melanoma, colorectal carcinoma, and glioblastoma by decreasing the number of GARP^+^LAP^+^ Tregs [91].

Albeit the role that GARP plays on cancer cells and on Tregs has been carefully studied, more research is required to understand how the cancer cells induce GARP expression and influence its function. The only stimuli described until now that trigger surface GARP are the BCR and TLRs engagement on human CD19^+^CD20^+^ B cells [25] (Caroline Wallace and Zihai Li, unpublished). This finding in combination with the study on the regulation of GARP promoter [32] might suggest that GARP expression requires the engagement of the MyD88 and NF-κB pathway. It is well known that chronic inflammation sets the stage for cancer development by triggering NF-κB signaling in immune and non-immune cells [92]. In this regard, it is interesting to notice that many cancers upregulate surface receptors to amplified NF-κB signaling as a surviving mechanism. For instance, in ovarian cancer, TLR2 [93] and lysophosphatidic acid (LPA) receptor [94] are associated with cancer stem cell renewal and cell invasion, respectively. The sustained NF-κB signaling might be one mechanism exploited by cancer cells to induce surface GARP expression. However, this hypothesis needs to be tested.

## Platelet GARP

As mentioned previously, GARP protein was first identified on activated Tregs and platelets [15]. Despite the increasing knowledge about the role of GARP in Tregs, little attention has been paid until recently to the role of GARP on platelets [24]. It is not entirely clear whether GARP plays a role in platelet activation and function since two different studies in two different animal models describe conflicting findings. The first study performed in *Danio rerio* (zebrafish) demonstrated that GARP is important for thrombus initiation and hemostasis; knockdown of the *Lrrc32* gene resulted in increased spontaneous bleeding events [95]. A second study, performed in a genetic mouse model where *Lrrc32* is specifically knocked out from platelets and megakaryocytes, shows that GARP is not necessary for thrombus formation and clot retraction, which is also confirmed by our own unpublished observations. Interestingly this last study shows that ex vivo platelet activation triggers increase in GARP surface expression, indicating that GARP might play a role in activating platelets [35]. Using the same platelet-specific GARP knockout mouse model, we recently demonstrated that GARP enhances the activation of latent TGF-β released by platelets [24]. Serum active TGF-β was drastically reduced in these mice, and interestingly, the similar phenotype was not observed in mice with platelet-specific deletion of *Tgfb1* gene, indicating that platelet GARP activates latent TGF-β1 secreted by cells other than by platelets [24]. Importantly, the same study demonstrated that the platelets contribute dominantly to the activity of TGF-β in the tumor environment. Among platelet-derived soluble factors, TGF-β is one of the main mediators for the platelet-dependent tumor growth [96, 97], which was once again confirmed by an unbiased biochemical and biophysical strategy [24]. Accordingly, platelet-specific deletion of GARP potentiated protective immunity against both murine models of melanoma and colon cancer [24]. This study also provides a mechanistic explanation as to why thrombocytosis is consistently associated with poor outcome in cancer [98]. Intriguingly, blocking platelet activation pharmacologically with aspirin and clopidogrel was shown to significantly enhance the adoptive T cell therapy of murine melanoma [24], which correlated with increased persistence and functionality of transferred donor T cells. This study lays a strong foundation for combining anti-platelet agents and immunotherapy as a novel strategy for cancer care in the future.

Beyond TGF-β, each activated platelet releases up to 80 α-granules secreting platelet-derived growth factors (PDGFs) in the tumor proximity which contribute to the platelet-cancer interaction [96, 99]. Activated platelets, for example, secrete vascular endothelial growth factor (VEGF) that induces angiogenesis and cell migration [100]. Additionally, multiple inflammatory cytokines are released by activated platelets such as IL-1, IL-6, granulocyte macrophage colony stimulating factor (GM-CSF) [101], and CD40L [102]. It is not known whether GARP plays a role in the release and/or activation of any other growth factor or cytokines produced by active platelets other than TGF-β, this intriguing possibility deserves further investigation.

## Conclusions and future perspective

Cancer immunotherapy, including the PD-1/PD-L1 immune checkpoint-targeted strategy, represents an exciting paradigm shift in oncology which aims to treat the immune system and not the cancer per se [103, 104]. A combination strategy with checkpoint inhibitors and other immune intervention is being pursued to overcome the resistance and lack of responsiveness observed in a majority of patients. GARP is a docking receptor for latent TGF-β and is involved in its activation. The function of GARP-TGF-β in Treg biology has been a topic of increasing interest. More recently, the tolerogenic roles of GARP expression by cancer cells and platelets have gained attention due to the belief that GARP represents an attractive target, either alone or in combination with immune checkpoint blockers, for cancer immunotherapy. Blocking the GARP-latent TGF-β complex on Tregs represents two possible advantages compared to the other strategies that either block TGF-β [105–107] or deplete Tregs with anti-CD25 antibody [108]. Blocking TGF-β systemically could cause undesired consequences due to the pleiotropic roles of this cytokine. GARP is mainly expressed by tumor cells, Tregs, and platelets present in the TME. Thus, targeting GARP has the advantage of blocking TGF-β activation only where it plays a pro-tumorigenic role. Since effector T cells express CD25 but not GARP, GARP-based depletion of Tregs shall be more advantageous over CD25-targeted approach [109]. However, any GARP-targeted strategy must be preceded with caution to not compromise the number and function of platelets.

For GvHD, reactive airway diseases or other inflammatory conditions, soluble GARP might find its role as a novel therapeutic agent. The full potential of the GARP-targeted strategy awaits more fundamental studies of GARP biology in relation to its biochemistry, cancer biology, and immunology. The strategic importance for GARP to be expressed by platelets in immune tolerance calls for further study of platelets, the last cellular entity in the blood to be discovered, in a wide range of diseases including their underappreciated roles in cancer immune evasion.

## Abbreviations

CTL: Cytotoxic T lymphocyte
FoxP3: Forkhead box P3
GARP: Glycoprotein-A repetitions predominant
GvHD: Graft versus host disease
LAP: Latency-associated peptide
LRRC32: Leucine-rich repeat-containing protein 32
LTBP: Latent TGF-β-binding protein
PDGFs: Platelet-derived growth factors
TAM: Tumor-associated macrophage
TCR: T cell receptor
TGF-β: Transforming growth factor beta
Th: T helper cell
TME: Tumor microenvironment
Treg: Regulatory T cell
αVβ: Alpha V beta integrins
